# Supplementary material for: Highly efficient all-inorganic perovskite solar cells with suppressed non-radiative recombination by a Lewis base
Source: Nat Commun. 2020 Jan 10;11:177. doi: 10.1038/s41467-019-13909-5 (PMC6954256; doi:10.1038/s41467-019-13909-5)
Supplement: Supplementary file 1 — Supplementary Information [file 41467_2019_13909_MOESM1_ESM.docx]

Supplementary Information

**Highly Efficient All-inorganic Perovskite Solar Cells with Suppressed Non-radiative Recombination by a Lewis Base**

Jing Wang^1†^, Jie Zhang^1†^, Yingzhi Zhou^2^, Hongbin Liu^4^, Qifan Xue^2^, Xiaosong Li^4^, Chu-Chen Chueh^5^, Hin-Lap Yip^2^*, Zonglong Zhu^1^*, Alex K. Y. Jen^1,3,4^*

J. Wang, Dr. J. Zhang, Prof. Z. L. Zhu, Prof. Alex K.-Y. Jen,

^1^ Department of Chemistry, City University of Hong Kong, Tat Chee Avenue, Kowloon, Hong Kong

Y. Yuan, Dr. Q. Xue, Prof. H.-L. Yip

^2^ Institute of Optoelectronic Materials and Devices, State Key Laboratory of Luminescent Materials and Devices, South China University of Technology, Guangzhou, P. R. China

Prof. Alex K.-Y. Jen

^3^Department of Materials Science and Engineering, City University of Hong Kong, Kowloon, 999077, Hong Kong

Dr. Hongbin Liu, Prof. X. Li, Prof. Alex K.-Y. Jen

^4^ Department of Chemistry, University of Washington, Seattle, WA 98195, USA

Prof. C.-C. Chueh

^5^Department of Chemical Engineering, National Taiwan University, Taipei 10617, Taiwan

^†^These authors contributed to this work equally.

*Corresponding authors: [zonglzhu@cityu.edu.hk](mailto:zonglzhu@cityu.edu.hk); alexjen@cityu.edu.hk; msangusyip@scut.edu.cn


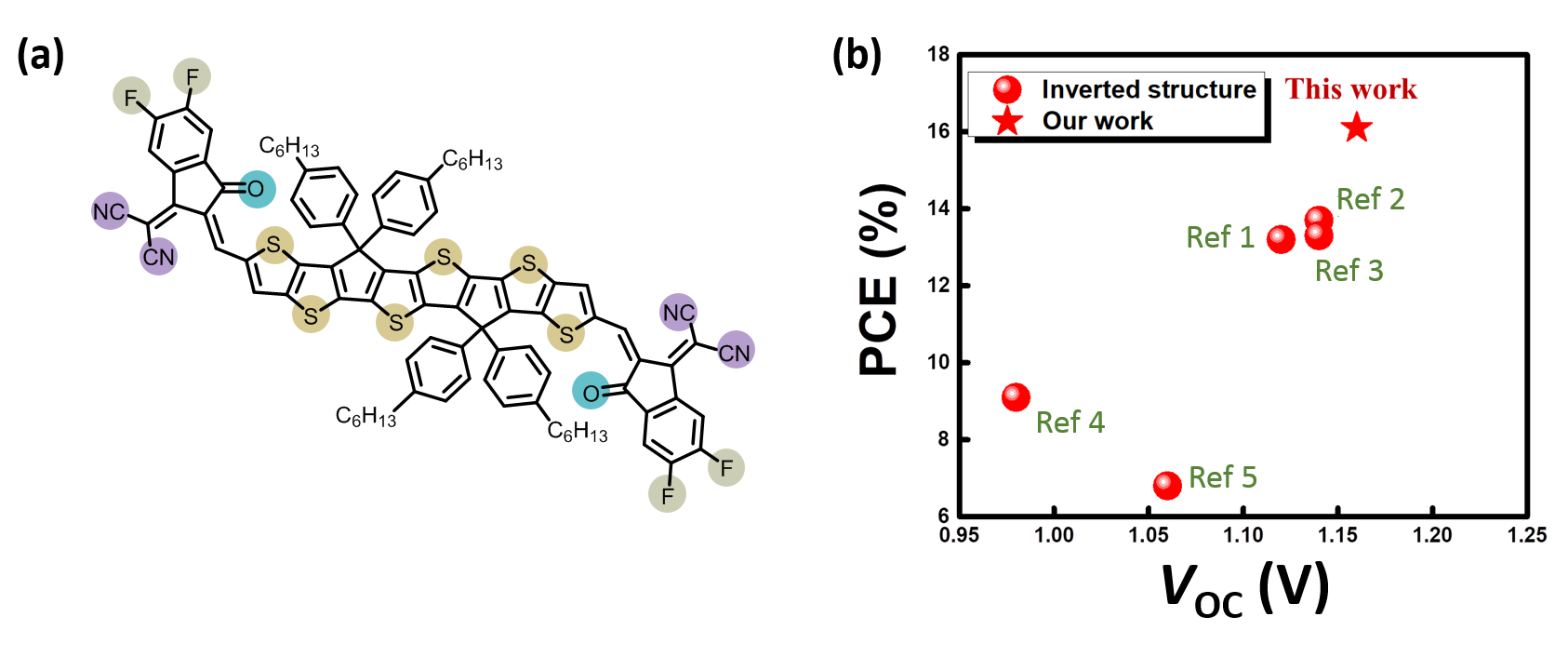


**Supplementary Figure 1.** Illustration of chemical structure of 6TIC-4F (a) and the *V*_OC_ and PCE of recently published all-inorganic PVSCs based on inverted structure in this work and in the literatures (b) (details in Table S1).


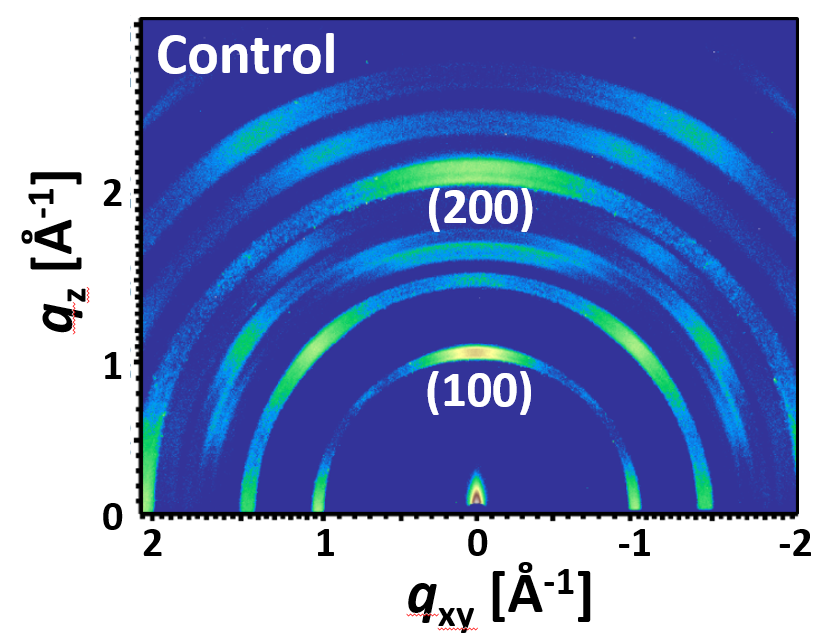


**Supplementary Figure 2.** Grazing incidence wide-angle X-ray scattering (GIWAXS) of inorganic CsPbBr_x_I_3-x_ film without 6TIC-4F treatment.


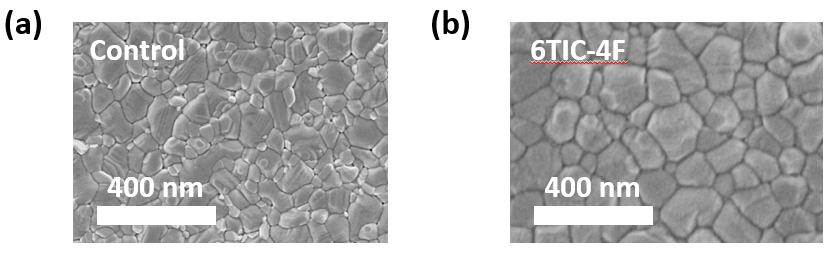


**Supplementary Figure 3.** SEM images of CsPbI_x_Br_3-x_ films (a) without and (b) with 6TIC-4F.


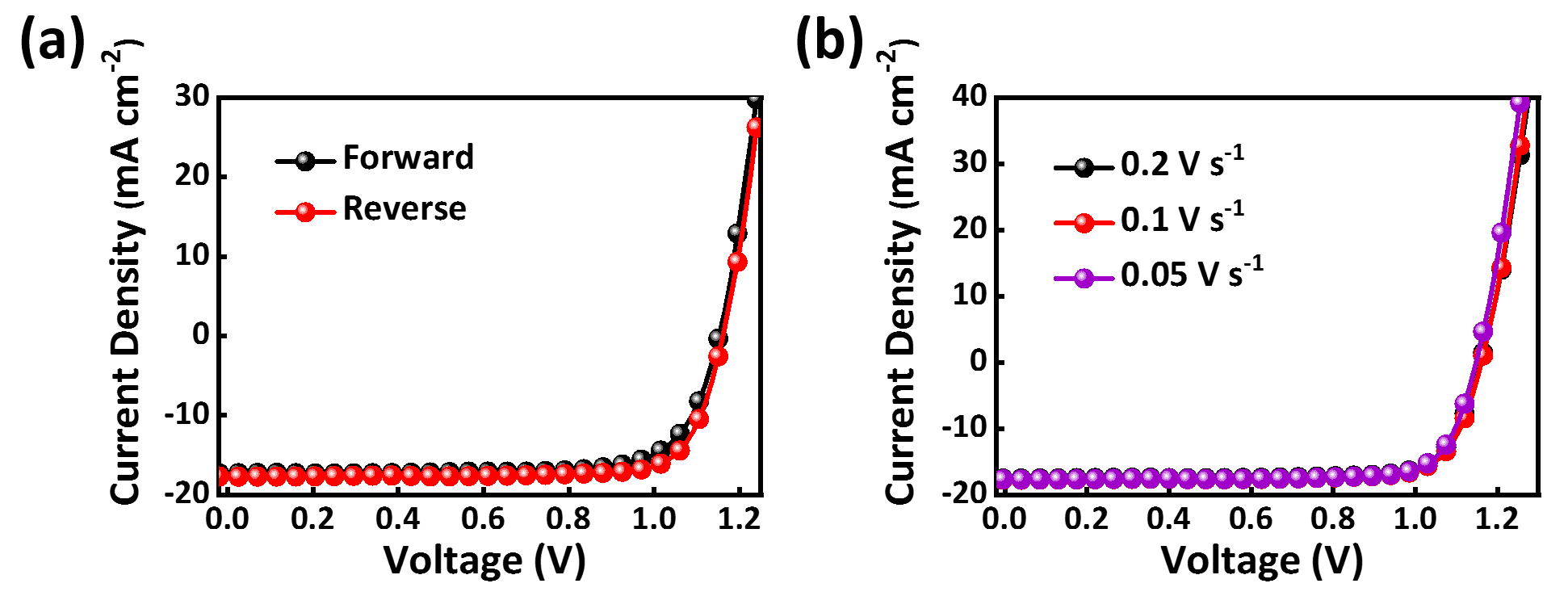


**Supplementary Figure 4.** (a) The *J-V* curves of CsPbI_x_Br_3-x_ PVSC under forward and reverse scan. (b) The *J-V* curves of CsPbI_x_Br_3-x_ PVSC under scan rate of 0.2, 0.1 and 0.05 V s^-1^.


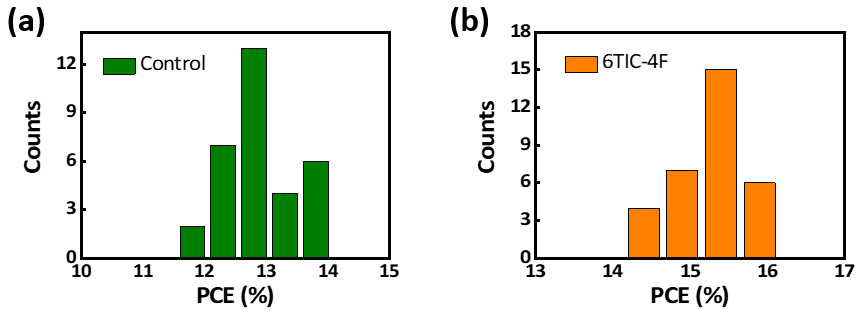


**Supplementary Figure 5.** The PCE histogram of CsPbI_x_Br_3-x_ PVSCs (a) without and (b) with 6TIC-4F passivation.


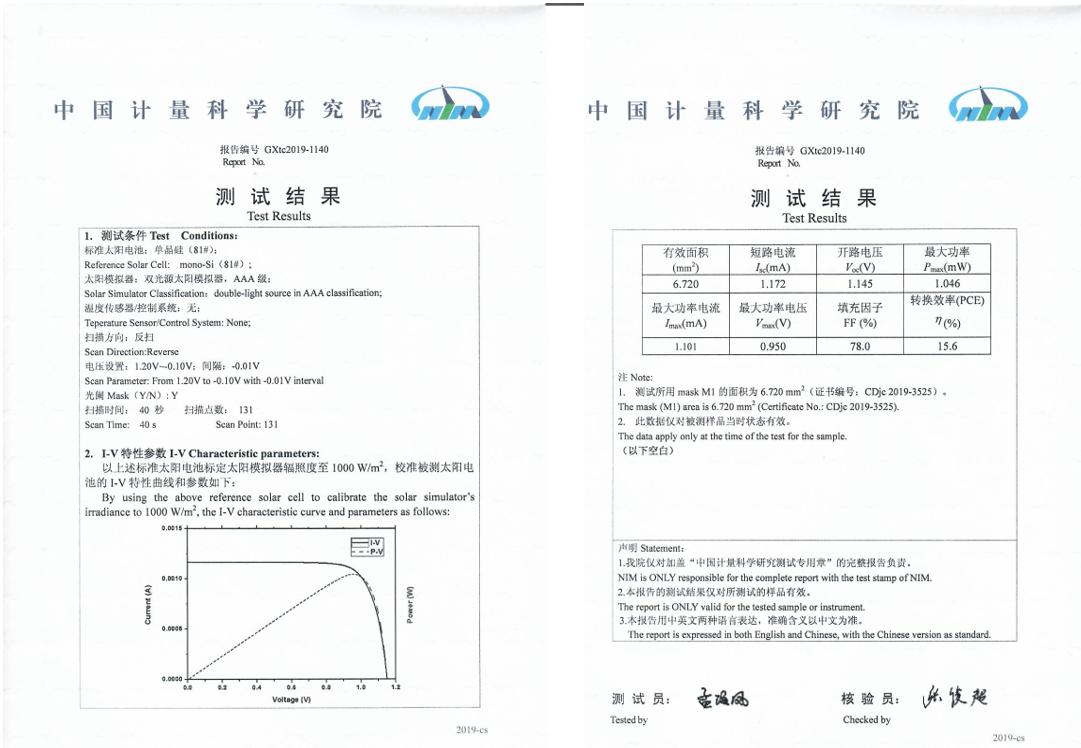


**Supplementary Figure 6.** The NIM certification files of our champion CsPbI_x_Br_3-x_ PVSC.


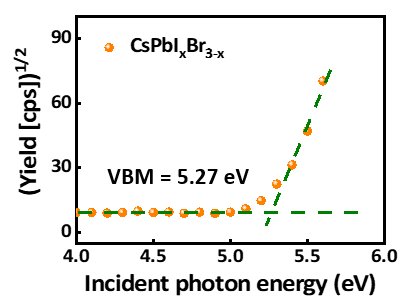


**Supplementary Figure 7.** The UPS spectrum of CsPbI_x_Br_3-x_ film. The valence band maximum (VBM) is 5.27 eV.


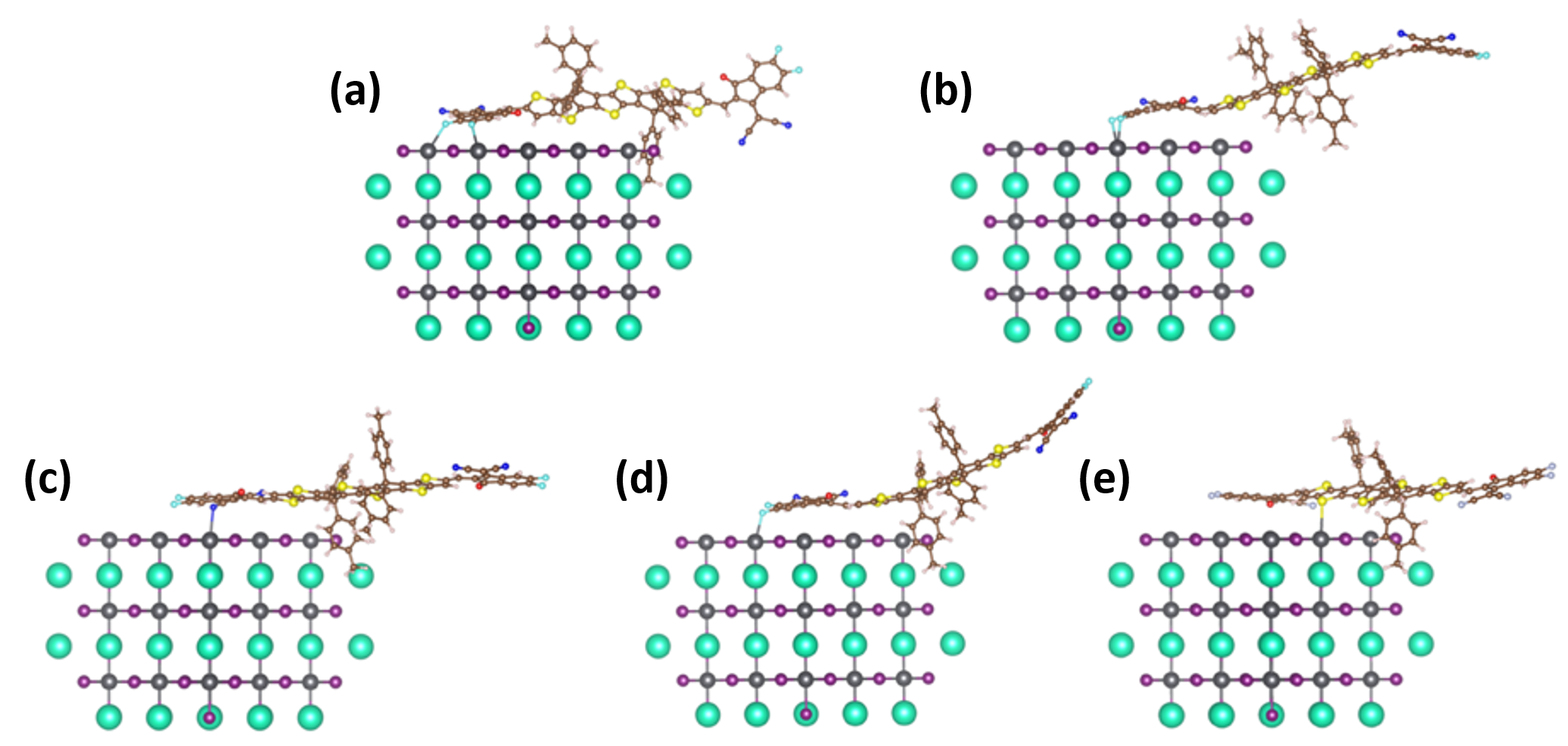


**Supplementary Figure 8.** The optimized structures of docking positions of 6TIC-4F on the uncompensated Pb surface, allowing fully relaxation of the 6TIC-4F molecules. The 6TIC-4F surface density is about 0.003 Å^-2^. Only F-Pb, N-Pb and S-Pb bonds stably formed after the geometry optimization. The formation energies of the five motifs are -2.90 eV, -2.81 eV, -3.14 eV, -2.20 eV, and -3.61 eV respectively for **Figure S8a**-**e**.


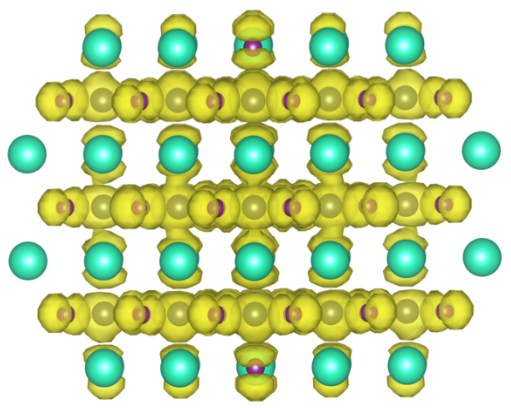


**Supplementary Figure 9.** The valence electron density of the Pb compensated pristine Cs_56_Pb_27_I_108_ cluster.


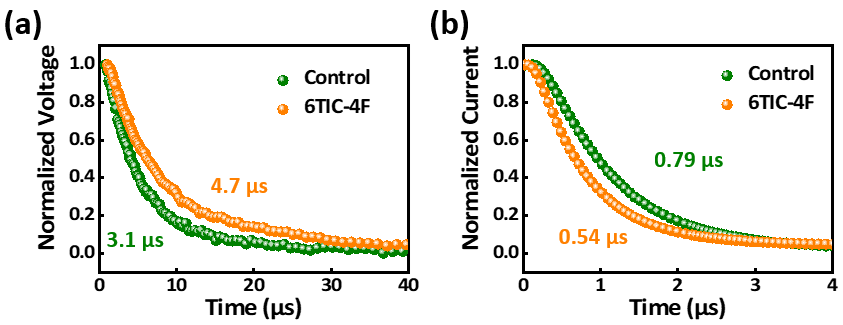


**Supplementary Figure 10.** The (a) TPV and (b) TPC curves of the CsPbI_x_Br_3-x_ PVSC without and with 6TIC-4F passivation.


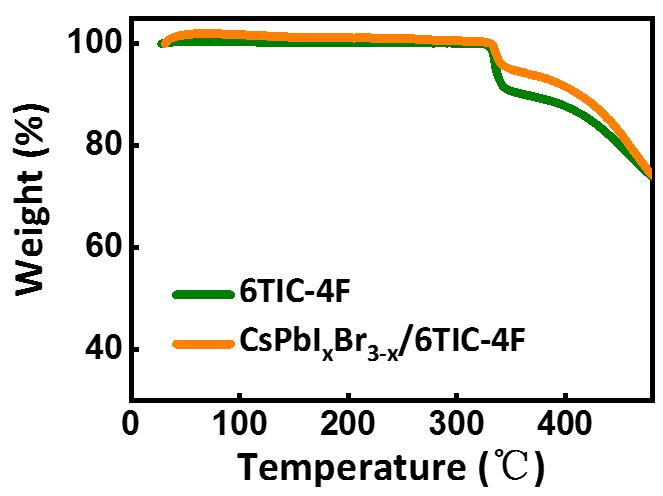


**Supplementary Figure 11.** The thermal gravimetric analysis (TGA) curves of 6TIC-4F and perovskite/6TIC-4F from room temperature to 600 °C.


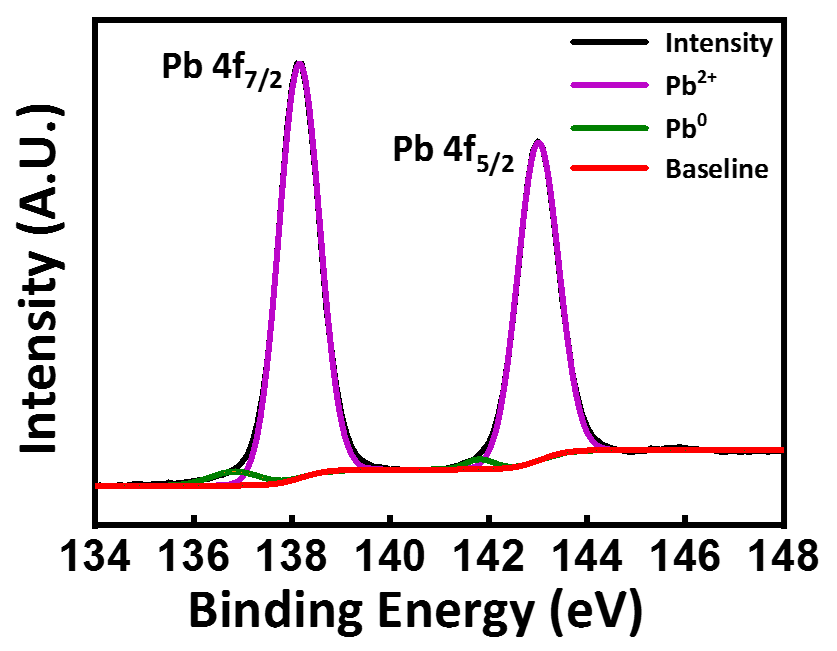


**Supplementary Figure 12.** The XPS signals of Pb states in CsPbI_x_Br_3-x_ film.


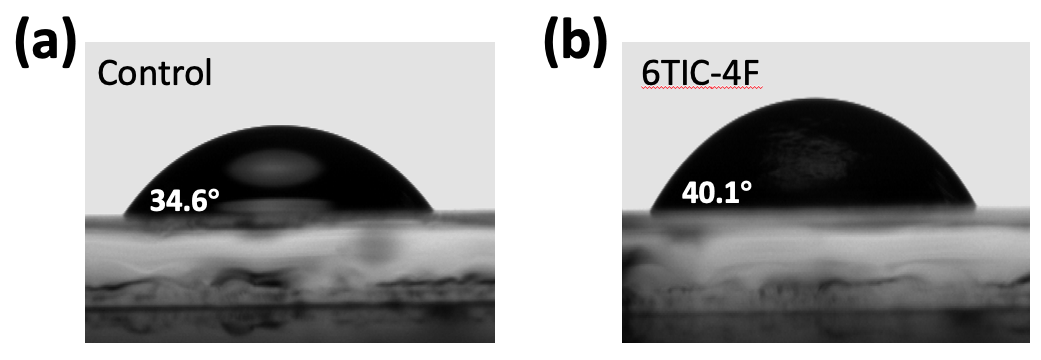


**Supplementary Figure 13.** Isopropanol contact-angle measurements of CsPbI_x_Br_3-x_ films without (a) and with (b) 6TIC-4F treatment.


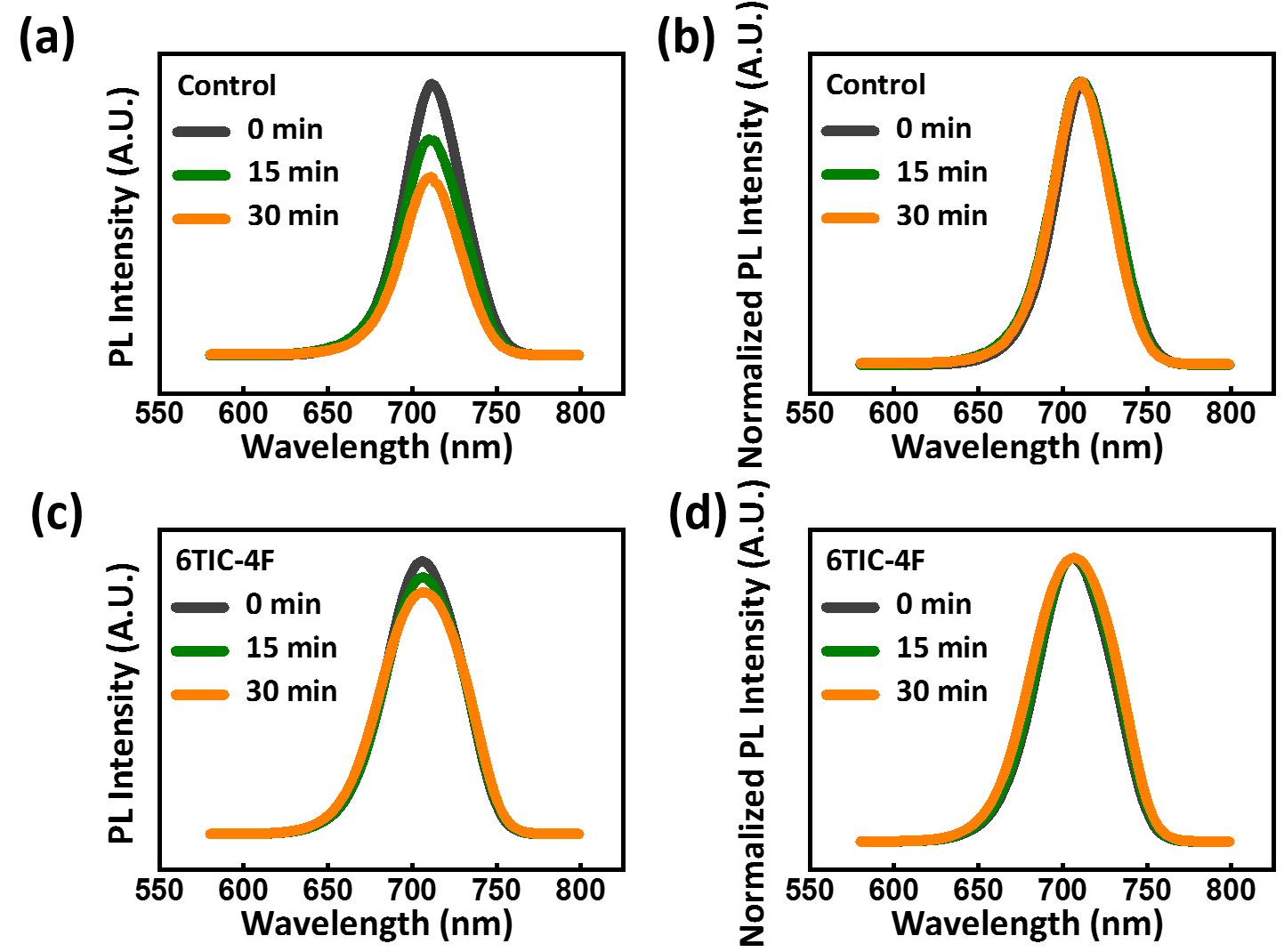


**Supplementary Figure 14.** The PL and normalized PL spectra of CsPbI_x_Br_3-x_ films without (a, b) and with (c, d) 6TIC-4F treatment under continuous one sun equivalent illumination.


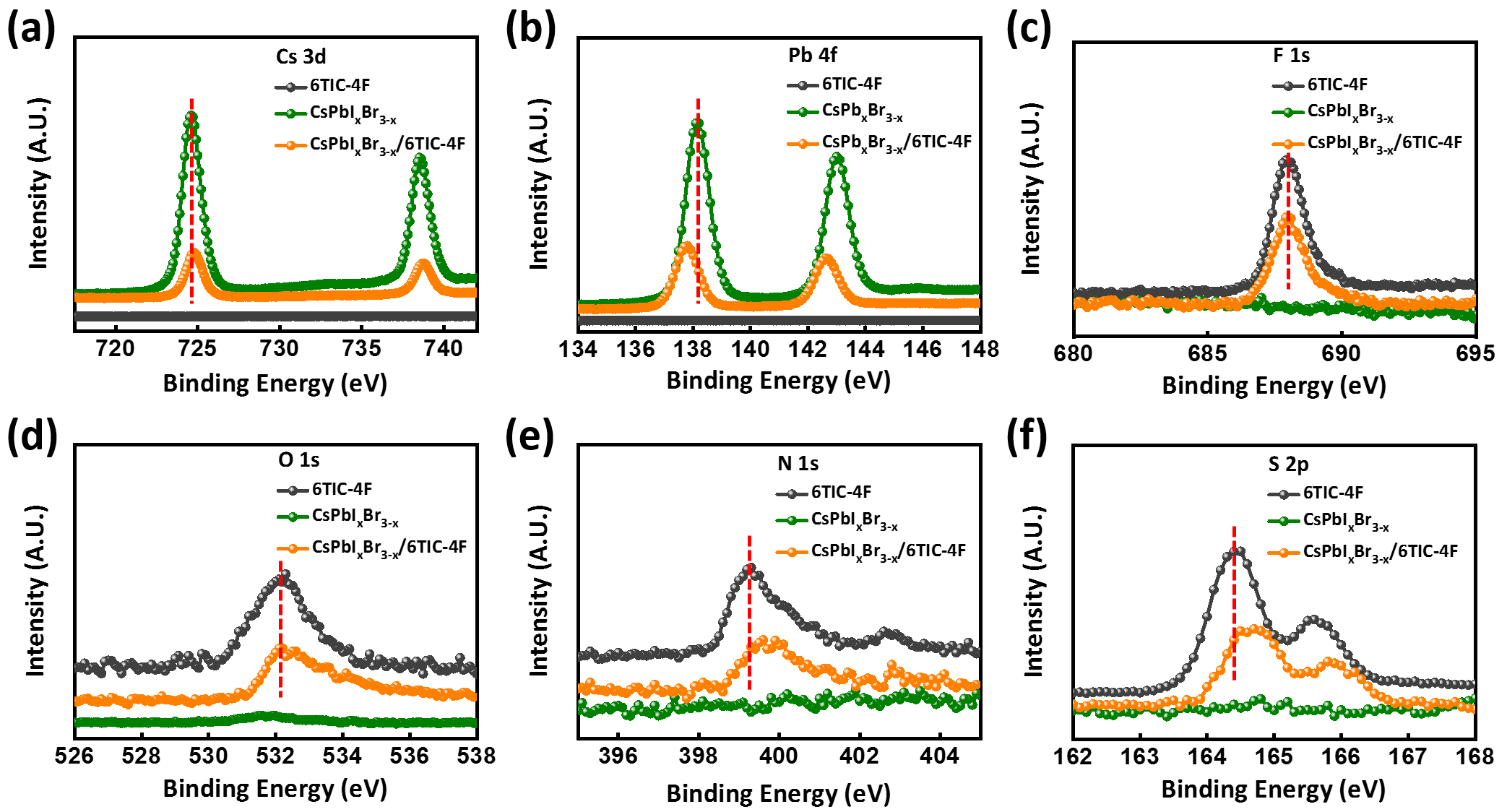


**Supplementary Figure 15.** XPS feature spectra of Cs 1d (a), Pb 4f (b), F 1s (c), O 1s (d), N 1s (e) and S 2p (f) for 6TIC-4F film, CsPbI_x_Br_3-x_ film and CsPbI_x_Br_3-x_/6TIC-4F film.


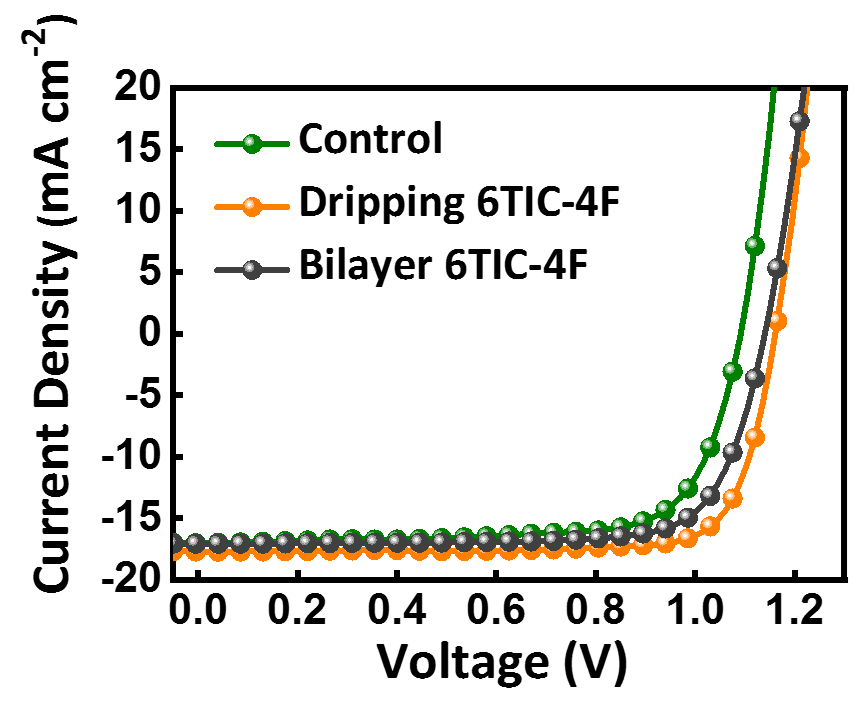


**Supplementary Figure 16.** The *J-V* curves of control device, control device with bilayer 6TIC-4F and device with dripping 6TIC-4F.

**Supplementary Table 1:** A summary of the detail performance parameters of inverted Cs-based inorganic solar cells reported recently.

| **Perovskite** | **Deposition method &**  **atmosphere** | **Device structure** | ***V*oc**  **(V)** | **PCE**  **(%)** | **Ref.** |
| --- | --- | --- | --- | --- | --- |
| **CsPbI_3_** | **Solution & Air-free** | **FTO /PTAA/ PVSC /PCBM/BCP/Ag** | **1.12** | **13.2** | [**^1^**](#_ENREF_1) |
| **CsPbI_2_Br** | **Solution & Air-free** | **FTO/NiOx/PVSC/ZnO@C_60_/Ag** | **1.14** | **13.7** | [**^2^**](#_ENREF_2) |
| **CsPbI_2_Br** | **Solution & Air-free** | **FTO/NiOx/PVSC/ZnO@C_60_/Ag** | **1.14** | **13.3** | [**^3^**](#_ENREF_3) |
| **CsPbI_2_Br** | **Solution & Air-free** | **FTO/NiMgLiO/ PVSC /PCBM/BCP/Ag,** | **0.98** | **9.1** | [**^4^**](#_ENREF_4) |
| **CsPbI_2_Br** | **Solution & Air-free** | **ITO/PEDOT:PSS/PVSC/PCBM/BCP/Al** | **1.06** | **6.8** | [**^5^**](#_ENREF_5) |
| **CsPbBr_x_I_3-x_** | **Solution & Air-free** | **ITO/ NiO_x_/ PVSC / ZnO/ C60/ Ag** | **1.16** | **16.1** | [**our**](#_ENREF_14) **work** |

**Supplementary Table 2:** The performance parameters of control device, control device with bilayer 6TIC-4F and device with dripping 6TIC-4F.

| **Devices** | ***V*_OC_**  **(V)** | ***J*_SC_**  **(mA cm^-2^)** | ***FF***  **(%)** | **PCE**  **(%)** |
| --- | --- | --- | --- | --- |
| **Control** | **1.10** | **17.0** | **74.2** | **13.9** |
| **Dripping 6TIC-4F** | **1.16** | **17.7** | **78.6** | **16.1** |
| **Bilayer 6TIC-4F** | **1.14** | **17.1** | **76.5** | **14.9** |

**Supplementary Note 1: Calculation of *V*_OC_ Loss**

According to the reciprocity relationship between photovoltaic external quantum efficiency ($\mathrm{EQE}_{\mathrm{PV}}$) and electroluminescence (EL), the *V*_OC_ of a solar cell can be calculated with the equation listed below:

$V_{\mathrm{OC}}=\frac{k_{B}T}{q}\ln\left( \frac{J_{\mathrm{SC}}}{J_{0}} \right)\cdots\cdots\cdots\cdots\cdots\cdots\cdots\cdots\cdots\cdots\cdots\cdots\cdots$ (1)

where $q$ is element charge, $k_{B}$ is Boltzmann constant, $T$ is temperature, *J*_SC_ is short-circuit current, *J*_0_ is dark saturation current. The expressions of *J*_SC_ and *J*_0_ are given by:

$J_{\mathrm{SC}}=q\int_{0}^{\infty} \mathrm{EQE}_{\mathrm{PV}}\left( E \right)\phi_{AM1.5}\left( E \right)dE\cdots\cdots\cdots\cdots\cdots\cdots\cdots\cdots\cdots\cdots\cdots\cdots\cdots$ (2)

$J_{0}=\frac{q}{{EQE}_{\mathrm{EL}}}\int_{0}^{\infty} \mathrm{EQE}_{\mathrm{PV}}\left( E \right)\phi_{\mathrm{BB}}\left( E \right)dE\cdots\cdots\cdots\cdots\cdots\cdots\cdots\cdots\cdots\cdots\cdots\cdots\cdots$ (3)

$$\phi_{\mathrm{BB}}\left( E \right)=\frac{2\pi E^{2}}{h^{3}c^{2}}\frac{1}{\exp\left( \frac{E}{k_{B}T} \right)-1}$$

where $\mathrm{EQE}_{\mathrm{EL}}$ is EL external quantum efficiency, $\phi_{AM1.5}$ is solar cell radiative spectrum, $\phi_{\mathrm{BB}}$ is black-body radiative spectrum, $c$ is light speed in vacuum.

In Schokley-Queisser limit (S-Q limit): (1) The $\mathrm{EQE}_{\mathrm{PV}}$ is described with Heaviside step function, where $\mathrm{EQE}_{\mathrm{PV}}\left( E \right)=\left\{ \begin{aligned} 1, E\geq E_{g} \\ 0, E<E_{g} \end{aligned} \right.$; (2) only the photos with energy larger than bandgap ($E_{g}$) are absorbed; (3) all recombination is radiative ($\mathrm{EQE}_{\mathrm{EL}}=1$). Therefore, *J*_SC_ and *J*_0_ in S-Q limit are written as:

$J_{\mathrm{SC}}^{\mathrm{SQ}}=q\int_{E_{g}}^{\infty} \phi_{AM1.5}\left( E \right)dE\cdots\cdots\cdots\cdots\cdots\cdots\cdots\cdots\cdots\cdots\cdots\cdots\cdots$ (4)

$J_{0}^{\mathrm{SQ}}=q\int_{E_{g}}^{\infty} \phi_{\mathrm{BB}}\left( E \right)dE\cdots\cdots\cdots\cdots\cdots\cdots\cdots\cdots\cdots\cdots\cdots\cdots\cdots$ (5)

Therefore, *V*_OC_ in S-Q limit is:

$V_{\mathrm{OC}}^{\mathrm{SQ}}=\frac{k_{B}T}{q}\ln\left( \frac{J_{\mathrm{SC}}^{\mathrm{SQ}}}{J_{0}^{\mathrm{SQ}}} \right)\cdots\cdots\cdots\cdots\cdots\cdots\cdots\cdots\cdots\cdots\cdots\cdots\cdots$ (6)

Considering the assumption of S-Q limit, $V_{\mathrm{OC}}^{\mathrm{SQ}}$ can be degraded to *V*_OC_ with 3 loss components. The first *V*_OC_ loss component, ${\Delta V}_{\mathrm{OC}}^{\mathrm{SC}}$, is due to the non-ideal ${EQE}_{\mathrm{PV}}$, which is less than 100%. In this condition, short-circuit current is expressed as:

$J_{\mathrm{SC}}=q\int_{0}^{\infty} \mathrm{EQE}_{\mathrm{PV}}\left( E \right)\phi_{AM1.5}\left( E \right)dE\cdots\cdots\cdots\cdots\cdots\cdots\cdots\cdots\cdots$ (7)

${\Delta V}_{\mathrm{OC}}^{\mathrm{SC}}$ is calculated as:

${\Delta V}_{\mathrm{OC}}^{\mathrm{SC}}=V_{\mathrm{OC}}^{\mathrm{SQ}}-\frac{k_{B}T}{q}\ln\left( \frac{J_{\mathrm{SC}}}{J_{0}^{\mathrm{SQ}}} \right)=\frac{k_{B}T}{q}\ln\left( \frac{J_{\mathrm{SC}}^{\mathrm{SQ}}}{J_{\mathrm{SC}}} \right)\cdots\cdots\cdots\cdots\cdots\cdots\cdots\cdots\cdots\cdots$ (8)

The second *V*_OC_ loss component comes from the energy loss associated with extra thermal radiation of solar cell in dark. In experiment, the $\mathrm{EQE}_{\mathrm{PV}}$ extends into the sub-bandgap region, where the black-body radiation increases with the photo energy lowering. Thus, this sub-bandgap $\mathrm{EQE}_{\mathrm{PV}}$ increased the dark saturation current. The dark saturation current in this condition is written as:

$J_{0}^{\mathrm{rad}}=q\int_{0}^{\infty} \mathrm{EQE}_{\mathrm{PV}}\left( E \right)\phi_{\mathrm{BB}}\left( E \right)dE\cdots\cdots\cdots\cdots\cdots\cdots\cdots\cdots\cdots\cdots$ (9)

therefore, the radiative *V*_OC_ loss, ${\Delta V}_{\mathrm{OC}}^{\mathrm{rad}}$, is:

${\Delta V}_{\mathrm{OC}}^{\mathrm{rad}}=\frac{k_{B}T}{q}\ln\left( \frac{J_{\mathrm{SC}}}{J_{0}^{\mathrm{SQ}}} \right)-\frac{k_{B}T}{q}\ln\left( \frac{J_{\mathrm{SC}}}{J_{0}^{\mathrm{rad}}} \right)=\frac{k_{B}T}{q}\ln\left( \frac{J_{0}^{\mathrm{rad}}}{J_{0}^{\mathrm{SQ}}} \right)\cdots\cdots\cdots\cdots\cdots\cdots\cdots\cdots\cdots$ (10)

The third *V*_OC_ loss component,${\Delta V}_{\mathrm{OC}}^{\mathrm{nonrad}}$, is ascribed to the non-radiative recombination in device, which can be calculated as:

${\Delta V}_{\mathrm{OC}}^{\mathrm{nonrad}}=\frac{k_{B}T}{q}\ln\left( \frac{J_{\mathrm{SC}}}{J_{0}^{\mathrm{rad}}} \right)-V_{\mathrm{OC}}\cdots\cdots\cdots\cdots\cdots\cdots\cdots\cdots\cdots\cdots\cdots$ (11)

According to **Equation 3** and S9, $J_{0}^{\mathrm{rad}}={\mathrm{EQE}_{\mathrm{EL}}\cdot J}_{0}$, so combining **Equation 1**, **Equation 11** can be rewritten as:

${\Delta V}_{\mathrm{OC}}^{\mathrm{nonrad}}=\frac{k_{B}T}{q}\ln\left( \frac{J_{\mathrm{SC}}}{{\mathrm{EQE}_{\mathrm{EL}}\cdot J}_{0}} \right)-\frac{k_{B}T}{q}\ln\left( \frac{J_{\mathrm{SC}}}{J_{0}} \right)$

$=-\frac{k_{B}T}{q}\ln\left( \mathrm{EQE}_{\mathrm{EL}} \right)$ $\cdots\cdots\cdots\cdots\cdots\cdots\cdots\cdots\cdots\cdots\cdots\cdots\cdots\cdots\cdots$ (12)

**Supplementary Reference**

1 Wu, T. et al. Efficient and Stable Cspbi3 Solar Cells Via Regulating Lattice Distortion with Surface Organic Terminal Groups. *Advanced Materials* **31**, 1900605 (2019).

2 Liu, C. et al. Structurally Reconstructed Cspbi2br Perovskite for Highly Stable and Square-Centimeter All-Inorganic Perovskite Solar Cells. *Advanced Energy Materials* **9**, 1803572 (2019).

3 Liu, C. et al. All-Inorganic Cspbi2br Perovskite Solar Cells with High Efficiency Exceeding 13%. *Journal of the American Chemical Society* **140**, 3825-3828 (2018).

4 Zhang, S. et al. Solvent Engineering for Efficient Inverted Perovskite Solar Cells Based on Inorganic Cspbi2br Light Absorber. *Materials Today Energy* **8**, 125-133 (2018).

5 Beal, R. E. et al. Cesium Lead Halide Perovskites with Improved Stability for Tandem Solar Cells. *The Journal of Physical Chemistry Letters* **7**, 746-751 (2016).
