# Supplementary material for: Highly efficient all-inorganic perovskite solar cells with suppressed non-radiative recombination by a Lewis base
Source: Nat Commun. 2020 Jan 10;11:177. doi: 10.1038/s41467-019-13909-5 (PMC6954256; doi:10.1038/s41467-019-13909-5)
Supplement: Supplementary file 3 — Reporting Summary [file 41467_2019_13909_MOESM3_ESM.pdf]

Corresponding author(s): Hin-Lap Yip, Zonglong Zhu and Alex K.-Y. Jen

Solar Cells Reporting Summary

Nature Research wishes to improve the reproducibility of the work that we publish. This form is intended for publication with all accepted papers reporting the characterization of photovoltaic devices and provides structure for consistency and transparency in reporting. Some list items might not apply to an individual manuscript, but all fields must be completed for clarity.

For further information on Nature Research policies, including our data availability policy, see Authors & Referees.

ü Experimental design

Please check: are the following details reported in the manuscript?

1. Dimensions

Item	Yes	No	Details
Area of the tested solar cells	☒	☐	The active area was 0.0672 cm2 (see "Methods" section in the manuscript).
Method used to determine the device area	☐	☒	The area of our masks was determined and certified at the National Institute of Metrology (NIM, China). However, we do not have the detailed information for the method.

2. Current-voltage characterization

Item	Yes	No	Details
Current density-voltage (J-V) plots in both forward and backward direction	☒	☐	Supplementary Figure 4a
Voltage scan conditions *For instance: scan direction, speed, dwell times*	☒	☐	See "Methods" section in the manuscript.
Test environment *For instance: characterization temperature, in air or in glove box*	☒	☐	See "Methods" section in the manuscript.
Protocol for preconditioning of the device before its characterization	☒	☐	See "Methods" section in the manuscript.
Stability of the J-V characteristic *Verified with time evolution of the maximum power point or with the photocurrent at maximum power point; see ref. 7 for details.*	☒	☐	Figure 2e

3. Hysteresis or any other unusual behaviour

Item	Yes	No	Details
Description of the unusual behaviour observed during the characterization	☒	☐	No unusual behaviour was observed and no significant hysteresis during characterization.
Related experimental data	☒	☐	Supplementary Figure 4

4. Efficiency

Item	Yes	No	Details
External quantum efficiency (EQE) or incident photons to current efficiency (IPCE)	☒	☐	Figure 2c
A comparison between the integrated response under the standard reference spectrum and the response measure under the simulator	☒	☐	Table 1
For tandem solar cells, the bias illumination and bias voltage used for each subcell	☐	☒	N.A.

5. Calibration

Item	Yes	No	Details
Light source and reference cell or sensor used for the characterization	☒	☐	See "Methods" section in the manuscript.

Confirmation that the reference cell was calibrated and certified

- [x] Yes
- [ ] No

See "Methods" section in the manuscript.

Calculation of spectral mismatch between the reference cell and the devices under test

- [x] Yes
- [ ] No

It has been calibrated

6. Mask/aperture

Size of the mask/aperture used during testing

- [x] Yes
- [ ] No

See "Methods" section in the manuscript.

Variation of the measured short-circuit current density with the mask/aperture area

- [x] Yes
- [ ] No

NIM certification report in Supplementary Figure 6

7. Performance certification

Identity of the independent certification laboratory that confirmed the photovoltaic performance

- [x] Yes
- [ ] No

The National Institute of Metrology (NIM, China)

A copy of any certificate(s)
Provide in Supplementary Information

- [x] Yes
- [ ] No

Supplementary Figure 6

8. Statistics

Number of solar cells tested

- [x] Yes
- [ ] No

Supplementary Figure 5

Statistical analysis of the device performance

- [x] Yes
- [ ] No

Supplementary Figure 5

9. Long-term stability analysis

Type of analysis, bias conditions and environmental conditions
For instance: illumination type, temperature, atmosphere humidity, encapsulation method, preconditioning temperature

- [x] Yes
- [ ] No

See "Device performance" Section in manuscript and Figure 2f
